# Use of long-lasting insecticidal nets among women attending antenatal clinic at a tertiary hospital in Bayelsa State, Nigeria 2019

**DOI:** 10.1186/s12936-020-03531-y

**Published:** 2020-12-14

**Authors:** Maria Imaobong Ibegu, Khadeejah Liman Hamza, Chukwuma David Umeokonkwo, Tamuno-Wari Numbere, Adolphe Ndoreraho, Tukur Dahiru

**Affiliations:** 1 Nigeria Field Epidemiology and Laboratory Training Programme, Abuja, Nigeria; 2 Department of Community Medicine, Alex Ekwueme Federal University Teaching Hospital Abakaliki, Ebonyi State, Nigeria; 3 Department of Community Medicine, Faculty of Clinical Sciences, Ahmadu Bello University, Zaria, Kaduna Nigeria

**Keywords:** Malaria, Long lasting insectidal nets, Antenatal care, Malaria in pregnancy, Nigeria

## Abstract

**Background:**

Malaria in pregnancy remains a major contributor to maternal and infant morbidity and mortality despite scale up in interventions. Its prevention is one of the major interventions in reducing maternal and infant morbidity and mortality. The ownership, utilization and predictors of use of long-lasting insecticide-treated nets (LLINs) for malaria prevention among women attending antenatal clinic (ANC) at a tertiary hospital in Bayelsa State Nigeria was assessed.

**Methods:**

A cross-sectional study of 297 women recruited through systematic sampling was carried out. Information on sociodemographic characteristics, ownership, source and utilization of LLINs, were collected with a pre-tested structured interviewer-administered questionnaire. The relationship between use of LLIN and sociodemographic characteristics was examined using chi square and logistic regression at 5% level of significance.

**Results:**

The mean age of respondents was 28.8 ± 2.6 years. Most (59.2%) had tertiary education and were mainly (88.2%) urban dwellers. Two hundred and fifty (84.2%) owned LLINs, and 196 (78%) used LLIN the night prior to the interview. Almost half of the respondents purchased their LLINs. Those who purchased LLINs were 3 times more likely to have used it (OR: 3.13, 95% CI 1.62–6.04) compared to those that got it free. Those who were gainfully employed (OR: 3.16, 95% CI 1.59–6.29) and those who earned above the minimum wage (OR: 2.88, 95% CI 1.45–5.72) were 3 times more likely to have used LLIN in their index pregnancy.

**Conclusion:**

The use of LLIN as a preventive measure against malaria was relatively high among the participants in this study, though still below national target. The major factors determining the use of LLIN among these women were purchase of LLINs and being gainfully employed. It was recommended that efforts should be made to enforce the policy of free LLINs at ANC registration at the tertiary hospitals, as this would further drive up ownership and utilization rates.

## Background

Malaria is a leading cause of death and disease in many developing countries, where children and pregnant women are the most affected. In 2018, malaria caused an estimated 228 million clinical episodes, and 405,000 deaths, with an estimated 94% of the deaths occurring in the WHO African region [1]. In sub-Saharan region, which includes Nigeria, the burden of malaria is largest, with over 90% of the world’s malaria-related deaths occurring therein. The *Anopheles gambiae* complex, a very efficient group of mosquitoes, is responsible for transmission of malaria in Africa, and the predominant parasite species in Africa is *Plasmodium falciparum.* This species causes severe malaria and death, and the local weather conditions in Africa allows transmission to occur year round [2]. In Bayelsa State, in a report by the State Ministry of Health, out of the 210 deaths that occurred from about 35 different diseases under the public health sector surveillance, malaria had the highest mortality with 102 deaths (49%) in 2011.

Protecting pregnant women is crucial in the fight against malaria, as malaria in pregnancy contributes significantly to deaths of mother and young children, estimated to amount each year to 10,000 women and up to 200,000 infants under one year of age [3]. Malaria in pregnancy contributes significantly to perinatal morbidity and mortality. It is known to cause higher rates of miscarriage, intrauterine death, premature delivery, low birth-weight babies, and neonatal deaths [4]. The knowledge and use of preventive methods against malaria is considered a cost-effective intervention in the fight against malaria, especially in endemic areas. It is also associated with significant reduction in malaria morbidity and mortality, particularly among pregnant women and children less than 5 years [5]. Malaria prevention and control in pregnant women in Africa is based on three pillars; long-lasting insecticidal nets (LLINs), intermittent preventive treatment in pregnancy (IPTp) with sulfadoxine-pyrimethamine, and effective case management of malaria illness and anaemia. However, coverage of these interventions is still suboptimal and continues to lag behind targets [6]. It has been shown that there are insufficient levels of access to and uptake of lifesaving malaria tools and interventions [7].

The last decade of malaria control has witnessed increased support by the government and partners in the areas of distribution of long-lasting insecticidal net (LLINs), replacement campaigns, intermittent preventive treatment (IPT), and a massive scale-up in malaria case management [7]. Between 2009 and 2013, the government of Nigeria, with support from several partners distributed approximately 52 million mosquito nets across the country. There was also a replacement campaign carried out between 2014 and 2015. In addition, programme efforts to fight malaria have emphasized the importance of net usage. This had led to a greater demand for mosquito nets.

Recognizing that mass campaigns of free LLINs are not enough to ensure high LLIN coverage over time, new continuous distribution strategies were developed to improve and maintain coverage in concert with campaigns. These strategies often focus on providing LLINs to the most vulnerable groups: pregnant women, infants and children [8]. One such strategy which is recommended by the WHO, is the combination of mass free net distribution through campaigns, and continuous distribution through multiple channels like antenatal care (ANC) clinics and the expanded program on immunization (EPI). The recommendations also state that continuous distribution through ANC and EPI channels should remain functional before, during and after mass distribution campaigns [9]. In many countries, public health-care providers require financial contributions from pregnant women who seek treatment for malaria. Such contributions can limit access to prevention and treatment services. Scarcity of resources is said to restrict the management of malaria in pregnancy and thereby hinder the achievement of optimal coverage from interventions. Even when health-care is free, transportation and other indirect costs can prevent pregnant women from seeking malaria prevention and treatment [6].

The levels of use of LLINs in the prevention of malaria was therefore assessed among pregnant women attending antenatal clinic in FMC Yenagoa.

## Methods

### Study setting

Bayelsa State is one of the states that made up the Niger Delta region of southern Nigeria. It shares boundaries with Delta State to the West and North, Rivers State to the East, and the Atlantic Ocean to the South. Bayelsa State is located in a tropical rainforest, with more than three quarters of the area covered by water. The area lies almost entirely below sea level, with a maze of meandering creeks and mangrove swamps. Bayelsa State has 2 tertiary hospitals: Federal Medical Centre, Yenagoa and Niger Delta University Teaching Hospital, Okolobiri.

This study was carried out in Federal Medical Centre Yenagoa (FMCY), Bayelsa State. FMCY is located in the capital city of Yenagoa. It attracts patients of diverse socio-economic status because of the high quality of services they offer, at a fair cost. FMCY offers antenatal care to pregnant women, with the clinic being manned by Consultant Obstetricians and resident doctors, on Monday through Thursday. Between 200 and 250 pregnant women are attended to at the antenatal clinic weekly. Presently, clients at the antenatal clinic have to procure drugs for IPTp on their own, and insecticide-treated nets are also currently not being distributed freely in antenatal clinics.

### Study design and population

A hospital based cross-sectional study among women attending antenatal care at FMC Yenagoa was conducted. Any pregnant woman attending antenatal clinic at Federal Medical Centre, Yenagoa was included. Those who were too ill to take part in the study were excluded.

### Sample size and sampling method

A sample size of 297 was calculated using a prevalence of 22.4% [10], at confidence level of 95%, and a precision of 5%. Pregnant women were recruited by systematic sampling until the desired sample size was reached. The sampling interval was determined by dividing the sample frame (total number of pregnant women in the antenatal clinic attendance register in FMCY within the month preceding the start of the study) by the sample size (297). According to the hospital report, the average patient flow for the antenatal clinic that month was 823. The *k*th value was 823/297 which is equal to 2.7, so every 3rd woman was asked, with the starting point determined randomly. Respondents were recruited daily from the antenatal clinics, which run on Monday through Thursday, over a duration of 3 months (March to May 2019).

### Data collection methods

A pre-tested structured interviewer administered questionnaire adapted from the MIS 2015 questionnaire [7], was used to collect data on sociodemographic characteristics of respondents, ownership/use of LLINs, as well as source of LLINs used. Pre-testing of the questionnaires was done at the Diete Koki Memorial Hospital, Yenagoa, a secondary health care facility that caters for both new and referred cases, in order to exclude ambiguities. Thereafter, identified problems in the questionnaire were addressed. The data collection was conducted by trained research assistants.

### Measurement of variables

The dependent variables for this study was ever use of LLIN by respondents which we defined it as having ever used LLIN in course of their pregnancy, while the independent variables were age, marital status, level of education, place of residence (urban or rural), parity, and income level.

Utilization rate was calculated using respondents who actually slept under an LLIN the night prior to the interview.

### Data analysis

Epi-Info version 7.3. and Microsoft Excel were used for the data analysis. We estimated the LLIN ownership and utilization. The relationship between the use of LLIN and sociodemographic characteristics was examined using Chi Square. To identify the predictors of use of LLIN, variables with p value of 0.1 or lower in bivariate analysis were included in the logistic regression model at 5% level of significance.

### Ethical consideration

An ethical approval to carry out the study was obtained from the Ethical review committee of Federal Medical Centre, Yenagoa (approval reference number: FMCY/REC/ECC/2019/JAN/149) A written informed consent was obtained from all respondents by way of signing or thumb printing on the informed consent form, after the nature and objectives of this study were explained to them. Participation was completely voluntary. The respondents’ right to refuse participation in the study was duly respected and was not allowed to affect the care they received in the health facility in any way. All information collected for the study was treated as confidential and stored in a computer with password protection.

## Results

The age of the respondents ranged between 23 and 37 years., with a mean age of 28.8 ± 2.6 years. Most women had tertiary level of education (59.2%), 35.7% had secondary education level, and the majority were traders (38.1%) and civil servants (37.7%). Respondents were either urban dwellers (88.2%) or rural dwellers (11.8%), all of whom were married (99%) or single (1%) as at time of study. Majority of participants were Ijaw (53.9%), followed by Igbo (23.9%), Yoruba (11.8%) and Hausa (4%, Table 1).

**Table 1:** Socio-demographic characteristics of women attending antenatal clinic in Federal Medical Centre, Yenagoa, Bayelsa State, 2019

Variable	Frequency (n = 297)	Percent (%)
Age (years)		
≤ 24	10	3.4
25–29	176	59.3
30–34	105	35.4
≥ 35	6	2.0
Mean age ± SD (years)	28.8 ± 2.6	
Education		
Primary	12	4.1
Secondary	106	35.7
Tertiary	176	59.2
No formal	3	1.0
Marital status		
Married	294	99.0
Single	3	1.0
Occupation		
Unemployed	55	18.5
Trading	113	38.1
Civil servant	112	37.7
Other^a^	17	5.7
Ethnicity		
Hausa	12	4.0
Igbo	71	23.9
Ijaw	160	53.9
Yoruba	35	11.8
Other	19	6.4
Place of residence		
Rural	35	11.8
Urban	262	88.2
Religion		
Christianity	283	95.3
Islam	14	4.7
Gravidity		
1	73	24.6
2–4	192	64.6
≥ 5	32	10.8
Monthly income		
≤ N18000	153	51.5
> N18000	144	48.5

^a^Tailor, hair stylist

Two hundred and fifty (84.2%) of participants mentioned using LLINs in the course of their current pregnancy, however 196 (78%) used it the night prior to the interview. About half of them (48.8%) were found to have purchased the LLINs from the market (Table 2).

**Table 2:** Long-lasting insecticidal net use among women attending antenatal clinic in Federal Medical Centre, Yenagoa, Bayelsa State, 2019

Practice	Frequency (n = 297)	Percent
Own LLIN		
Yes	250	84.2
No	47	15.8
Ever use of LLIN in current pregnancy		
Yes	250	84.2
No	*47*	*15.8*

	Frequency (n = 250)	Percent
Use of LLIN on the night prior to interview		
Yes	196	78.4
No	54	21.6
Source of LLIN		
Purchased	122	48.8
Received free	128	51.2

Those who were employed were 3 times more likely to have used LLIN, when compared to those who were not employed, and this was statistically significant [OR = 3.16 (CI 1.59—6.29)]. Those who earned above the minimum wage were nearly 3 times more likely to have used LLIN when compared to those who earned below the minimum wage (Table 3). This was statistically significant [OR = 2.88 (CI 1.45–5.72)]. Similarly, those who purchased LLINs were 3 times more likely to have used one [OR = 3.13 (CI 1.62–6.04)].

**Table 3:** Socio-demographic factors associated with use of long-lasting insecticidal net among women attending antenatal clinic in Federal Medical Centre, Yenagoa, Bayelsa State, 2019

Variables	Yes n (%)	No n (%)	Crude Odds Ratio (95% CI)
Use of ITN/LLIN			
Age category			
≤ 30 yrs	157 (83.5)	31 (16.5)	0.9 (0.60–2.21)
> 30 yrs	93 (85.3)	16 (16.7)	1
Level of Education			
Primary/secondary	100 (82.6)	21 (17.4)	1
Tertiary	150 (85.2)	26 (14.8)	1.2 (0.65–2.27)
Place of residence			
Rural	32 (91.4)	3 (8.6)	2.2 (0.14–1.58)
Urban	218 (83.2)	44 (16.8)	1
Occupation			
Employed	212 (87.6)	30 (12.4)	*3.16 (1.59–6.29)* *
Unemployed	38 (69.1)	17 (30.9)	1
Income			
> N18000	131 (90.9)	13 (9.1)	2.88 (1.45–5.72)*
≤ N18000	119 (77.8)	34 (22.2)	1
Source of LLIN			
Purchased	107 (87.7)	15 (12.3)	*3.13 (1.62–6.04)* *
Received free	89 (69.5)	39 (30.5)	1

* Statistically significant

There was statistically significant association between being employed and the use of LLIN [OR = 9.46 (CI 1.21–73.96)]. There was also a statistically significant association between purchase of LLIN and the use of LLIN [OR = 2.47 (CI 1.25–4.85)] (Table 4).

**Table 4:** Predictors of long-lasting insecticidal net use among women attending antenatal clinic in Federal Medical Centre, Yenagoa, Bayelsa State, 2019

Factors	Adjusted Odds ratio (AOR)	95% CI	p-value
Occupation			
Employed	9.46	1.21–73.96	0.032*
Unemployed	1		
Income			
> N18000	1.14	0.60–2.18	0.680
≤ N18000	1		
Source of LLIN			
Purchased	2.47	1.25–4.85	0.009*
Received free	1		

* Statistically significant

## Discussion

This study found that majority of respondents used LLIN as a measure to prevent malaria in the current pregnancy. Most of them had actually slept in one the night prior to the interview, giving a utilization rate of 78%, which was a similar finding to the utilization rates found in the studies by Mbonye et al*.* [11] and Boene et al*.* [12] but much higher than the utilization rates that was found in other studies [13–15]. Even though the ownership and use of LLIN by participants of this study was relatively high, it still falls below the national target of 100% and 80% respectively. The high educational level of respondents, and the fact that most of them resided in urban areas may have accounted for the high usage of LLINs by respondents.

Of the women who reported ownership of nets, almost half got them from the open market, shop, or supermarket. This made it difficult to establish the quality of nets being used. This was in contrast to studies carried out in Rwanda and Myanmar, where less than 1% and 25% of respondents respectively were found to have purchased their LLIN [16, 17]. A study found that the poorest socio-economic groups were less likely to purchase an LLIN, and also stated a lower willingness to pay for an LLIN [18]. Another study carried out in Madagascar found that distributing LLINs for free or at a small nominal price will maximize demand and effective coverage. It suggested that alternative sources of financing should be identified to completely (or almost completely) subsidize the cost of LLINs in order to maximize coverage of LLINs among poor populations at risk of malaria [19]. While many favor free distribution of LLINs, there are notable drawbacks. Free distribution requires a great deal of donor support and resources, it may therefore prove to be unsustainable due to high resource demand [20].

In this study, being employed and earning above the minimum wage were significantly associated with the use of LLIN by pregnant women. This may explain why half of our respondents who owned LLINs had to buy them, as it may not be possible to do so, if they did not have the required funds to do so.

In a study carried out in Ibadan, Nigeria, preventive behavior practiced by women was positively influenced by their level of education [21]. There was a similar finding in another study which found that women who were exposed to educational programs on the adverse effects of malaria in pregnancy were more likely to use insecticide treated nets [22]. Another research [23] found the main determinants associated with use of LLINs to be education level, household income, socio-economic status, malaria and LLIN knowledge, and urban residence. Similarly, in a study carried out in Ethiopia [24] age, educational status, occupation, income and husbands’ educational status were predictors of LLIN utilization. This study indicates that financially empowering women may help to promote their health and general wellbeing.

Being a hospital-based study, the use of questionnaires to obtain information from respondents may not have provided enough evidence of their actual practice. Despite the potential limitations of this study, the findings reveal that the policy on free distribution of LLINs is not effective, as a large number of study respondents are purchasing them.

## Conclusion

The use of LLIN as a preventive measure against malaria was relatively high among the participants in this study, though still below national target. The major factors determining the use of LLIN among these women was income above the minimum wage and being gainfully employed. It is recommended that efforts should be made to enforce the policy of free LLINs at ANC registration at the tertiary hospitals, as this would further drive up utilization rates.

## Data Availability

The data set used during this study are available from the corresponding author on reasonable request.

## Abbreviations

ANC: Antenatal care
EPI: Expanded programme on immunization
FMCY: Federal Medical Center, Yenagoa
IPTp: Intermittent Prophylactic Treatment in Pregnancy
LLINs: Long lasting insecticidal nets
WHO: World Health Organization

## References

[1] WHO (2019). World Malaria Report 2019.

[2] Centers for Disease Control and Prevention. Malaria. https://www.cdc.gov/malaria/malaria_worldwide/impact.html. Accessed 15 June 2018.

[3] United Nations Children’s Fund. Malaria fact sheet. https://www.unicef.org/media/media_81674.html. Accessed 15 June 2018.

[4] Schantz-Dunn J, Nour NM (2009). Malaria and pregnancy: a global health perspective. Rev Obstet Gynecol.

[5] Dawaki S, Al-Mekhlafi HM, Ithoi I, Ibrahim J, Atroosh WM, Abdulsalam AM (2016). Is Nigeria winning the battle against malaria ? Prevalence, risk factors and KAP assessment among Hausa communities in Kano State. Malar J.

[6] Rogerson SJ, Desai M, Mayor A, Sicuri E, Taylor SM, Van EAM (2018). Series Malaria in pregnancy 1 Burden, pathology, and costs of malaria in pregnancy : new developments for an old problem. Lancet Infect Dis.

[7] National Malaria Elimination Programme (NMEP), Commission(NPopC) NP, National Bureau Of Statistics (NBS), ICF International. Malaria Indicator Survey (MIS) 2015. Abuja; 2016.

[8] Theiss-Nyland K, Lines J, Fine P (2019). Can ITN distribution policies increase children ’ s ITN use? A DHS analysis. Malar J.

[9] WHO. Achieving and maintaining universal coverage with long-lasting insecticidal nets for malaria control. Geneva, World Health Organization, 2017. https://www.who.int/malaria/publications/atoz/who_recommendation_coverage_llin/en/. Accessed 3 September 2019.

[10] Ebenezer A, Stephen DP, Enaregha EB (2016). Patterns of *Plasmodium falciparum* malaria among pregnant women attending antenatal clinics in the communities along the Epie creek, Bayelsa State, Nigeria. Ann Biol Res.

[11] Mbonye AK, Mohamud SM, Bagonza J (2016). Perceptions and practices for preventing malaria in pregnancy in a peri-urban setting in south-western Uganda. Malar J.

[12] Boene H, González R, Valá A, Rupérez M, Velasco C, Machevo S (2014). Perceptions of malaria in pregnancy and acceptability of preventive interventions among Mozambican pregnant women: Implications for effectiveness of malaria control in pregnancy. PLoS One.

[13] Ezire O, Adebayo SB, Idogho O, Bamgboye EA, Nwokolo E (2015). Determinants of use of insecticide-treated nets among pregnant women in Nigeria. Int J Womens Health.

[14] Ordinioha B (2012). The use and misuse of mass distributed free insecticide-treated bed nets in a semi-urban community in Rivers State. Nigeria Ann Afr Med.

[15] Tijani A (2017). Malaria prevention practices among pregnant mothers in Osogbo, Nigeria. BMJ Glob Health.

[16] Kateera F, Ingabire CM, Hakizimana E, Rulisa A, Karinda P, Grobusch MP (2015). Long-lasting insecticidal net source, ownership and use in the context of universal coverage: a household survey in eastern Rwanda. Malar J.

[17] Nyunt MH, Aye KM, Kyaw MP, Kyaw TT, Hlaing T, Oo K (2014). Challenges in universal coverage and utilization of insecticide-treated bed nets in migrant plantation workers in Myanmar. Malar J.

[18] Onwujekwe O, Hanson K, Fox-Rushby J (2004). Inequalities in purchase of mosquito nets and willingness to pay for insecticide-treated nets in Nigeria: Challenges for malaria control interventions. Malar J.

[19] Comfort AB, Krezanoski PJ (2017). The effect of price on demand for and use of bednets: Evidence from a randomized experiment in Madagascar. Health Policy Plan.

[20] Sexton AR (2011). Best practices for an insecticide-treated bed net distribution programme in sub-Saharan eastern Africa. Malar J.

[21] Salami KK, Umego NL (2018). Household evironment and malaria in pregnancy in Ibadan, Nigeria. Health (Irvine Calif).

[22] Iliyasu Z, Gajida AU, Galadanci HS, Abubakar IS, Baba AS, Jibo AM (2012). Adherence to intermittent preventive treatment for malaria in pregnancy in urban Kano, northern Nigeria. Pathog Glob Health.

[23] Singh M, Brown G, Rogerson SJ (2013). Ownership and use of insecticide-treated nets during pregnancy in sub-Saharan Africa: a review. Malar J.

[24] Yitayew AE, Enyew HD, Goshu YA (2018). Utilization and associated factors of insecticide-treated bed net among pregnant women attending antenatal clinic of Addis Zemen Hospital, North-Western Ethiopia: an institutional based study. Malar Res Treat.

